# Underwater ultra-slim endoscopy for radiation-free intestinal stenting: overcoming difficult strictures

**DOI:** 10.1055/a-2724-7196

**Published:** 2025-11-05

**Authors:** Zhiwei Yang, Weiwei Liu, Sique Yu, Chunting Liu

**Affiliations:** 1 Department of Gastroenterology and Endoscopy Center, The Third Affiliated Hospital of Sun Yat-sen University, Yuedong Hospital, Meizhou, China

We previously developed a radiation-free self-expandable metal stent (SEMS) placement technique using an ultra-slim endoscope for malignant colonic obstruction 1 . However, in severe cases, it can be challenging to move the guidewire through the stenosis. Therefore, we developed an underwater ultra-slim endoscope technique utilizing reciprocal guidance between the guidewire and endoscope.

This report describes a 66-year-old male who presented with abdominal pain, distension, nausea, and vomiting. Computed tomography demonstrated an obstructive mass in the descending colon requiring SEMS placement ( Fig. 1 ). After gastrointestinal decompression and cleansing enema, colonoscopy showed severe stenosis with poor visualization ( Fig. 2 ). After thorough irrigation, we performed an endoscopy using the ultra-slim endoscope equipped with a water-infusion cap (Anrei Medical, AMH-AH-03, Fig. 3 ).

**Fig. 1:**
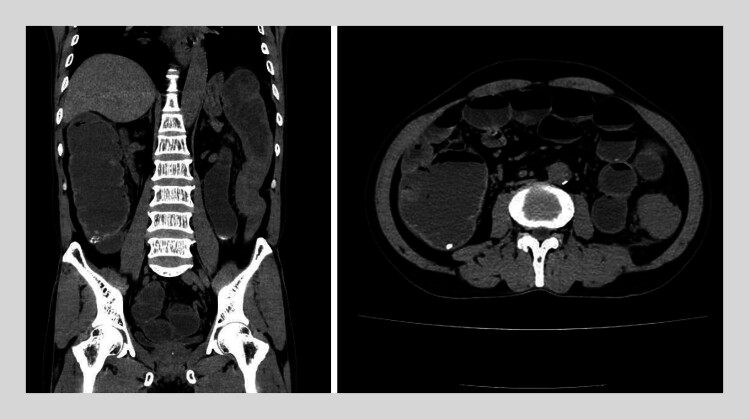
Computed tomography (CT) image of descending colon cancer. The CT image demonstrated wall thickening of the descending colon and dilation of the proximal colon and small intestine.

**Fig. 2:**
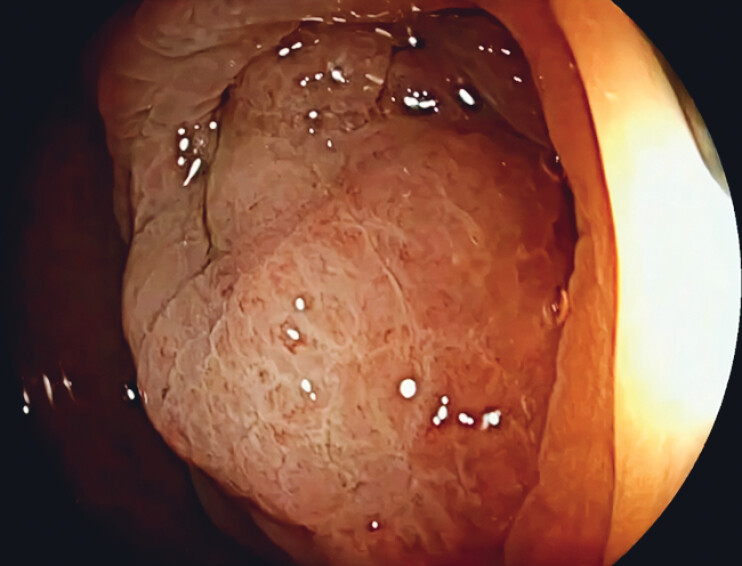
Endoscopic image of descending colon cancer. Marked luminal stenosis was present, with poor visualization of the entrance to the narrowed segment.

**Fig. 3:**
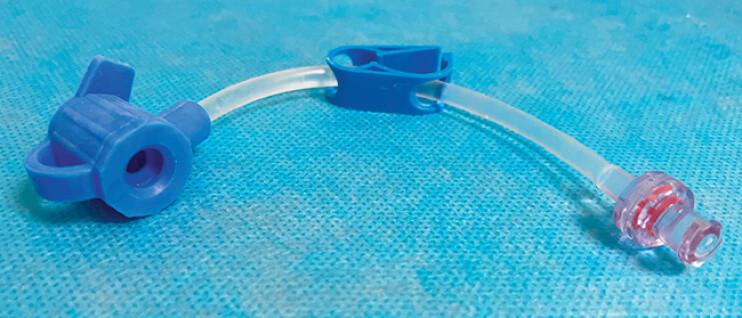
Endoscopic cap with water infusion function. After attachment, the ultra-slim endoscope was equipped with water infusion capability, allowing for underwater endoscopic procedures.

Our novel endoscopic technique offered multiple advantages in the management of this case. The ultra-slim, flexible endoscope facilitated access to the stricture and enabled precise guidewire manipulation. The underwater environment, combined with water jet irrigation, enhanced visualization by providing magnification and displacing the mucosa. The water infusion also reduced the gas-related risks and improved the visualization of the obstruction, which appeared as a “smoke-like” dispersion of fecal fluid ( Fig. 4 **a** ). The guidewire was successfully maneuvered through the stenosis and positioned within the dilated colon ( Fig. 4 **b** ), enabling successful deployment of the SEMS ( Fig. 4 **c** ).

**Fig. 4:**
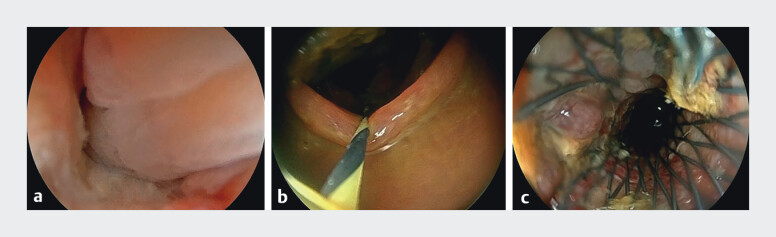
Endoscopic images of SEMS placement under water immersion. **a** The magnification and flushing effects of water provided a clear view of the entrance to the stenotic segment, facilitating guidewire insertion. **b** After the ultra-slim endoscope passed through the stenosis, a zebra guidewire was inserted into the dilated colon. **c** The image demonstrated successful SEMS deployment.

Overall, the underwater ultra-slim endoscope technique reduces the risk of complications and improves the identification of strictures. Compared to the double-scope colonoscope and other fluoroscopy techniques, it offers a simpler, more cost-effective, and radiation-free method for SEMS placement in patients with severe colonic obstruction 2 ( Video 1 ).

Underwater ultra-slim endoscopy for radiation-free self-expandable metal stent (SEMS) placement in a patient with severe malignant colonic obstruction.Video 1

Endoscopy_UCTN_Code_TTT_1AQ_2AF

## Notes

Endoscopy E-Videos https://eref.thieme.de/e-videos
*E-Videos* is an open access online section of the journal *Endoscopy* , reporting on interesting cases and new techniques in gastroenterological endoscopy. All papers include a high-quality video and are published with a Creative Commons CC-BY license. Endoscopy E-Videos qualify for HINARI discounts and waivers and eligibility is automatically checked during the submission process. We grant 100% waivers to articles whose corresponding authors are based in Group A countries and 50% waivers to those who are based in Group B countries as classified by Research4Life (see: https://www.research4life.org/access/eligibility/ ). This section has its own submission website at https://mc.manuscriptcentral.com/e-videos .

## References

[1] Yang Z Wang Z Yu S A novel radiation-free method for intestinal stent implantation: A case report Asian J Surgin press 10.1016/j.asjsur.2025.05.077

[2] Hu J Zheng J Yang C A radiation-free novel approach for intestinal stent placement: the “scope-in-scope” technique Endoscopy 2024 56 01 E313 E314 10.1055/a-2291-9315 38593996 PMC11003807

